# Radioembolization for Hepatocellular Carcinoma using TheraSphere^®^

**DOI:** 10.4103/1319-3767.80388

**Published:** 2011

**Authors:** Safiyya Mohamed Ali

**Affiliations:** Liver Diseases Research Center, College of Medicine, King Saud University, Riyadh, Saudi Arabia

**Keywords:** Efficacy, hepatocellular carcinoma, locoregional therapy, radioembolization, safety, therapy

## Abstract

**Background/Aim:**

Hepatocellular carcinoma (HCC) is the most common primary malignancy of the liver. Radioembolization with yttrium-90 (Y90) microspheres is a new concept in radiation therapy for HCC. This review focuses on the indications, efficacy, side effects, and future direction of Y90 therapy, using TheraSphere^®^, in HCC patients.

**Results:**

Comprehensive literature reviews have described the clinical and scientific evidence of Y90 therapy. The Radioembolization Brachytherapy Oncology Consortium has concluded that there is sufficient evidence to support the safe and effective use of this locoregional therapy in HCC patients, including those with portal vein thrombosis.

**Conclusions:**

There are currently no randomized clinical trials done on TheraSphere^®^ and none of the studies so far have shown a survival benefit. Thus, although it represents a very promising therapy with excellent initial results, it cannot be fully recommended yet, till well-designed, large, randomized clinical studies are conducted showing survival benefits.

Hepatocellular carcinoma (HCC) is the most common primary malignancy of the liver. It represents the sixth most common malignancy worldwide and the third most common cause of cancer-related mortality.[1,2] Resection and transplantation are the only curative treatments at present. However, the role of surgery is restricted. Resection can only be done in patients with normal liver function and transplantation is only possible in patients who satisfy the Milan criteria.[3] The only systemic chemotherapeutic drug that has shown some potential in managing HCC is Sorafenib.[4] The liver is also a common site for metastasis of other tumors. Surgical options are limited in the management of secondary liver tumors[5] and are usually managed by systemic chemotherapeutic agents. Unfortunately, many malignancies are not responsive to chemotherapy.

Locoregional therapies such as radiofrequency ablation and trans-arterial chemoembolization (TACE) are emerging as promising strategies for the management of liver tumors. These therapies are able to deliver the desired dose to the target with minimal toxicity to the system. Traditionally, the use of external beam radiation has been limited due to the sensitivity of the normal hepatic tissue.[6] Despite improvements in the delivery of focused external beams, some technical limitations remain.[7] Radioembolization with yttrium-90 (Y90) microspheres is a new concept in radiation therapy for HCC. Here, radio-labeled particles are injected through the hepatic artery, become trapped at the precapillary level and emit lethal internal radiation. This method limits exposure to the surrounding normal parenchyma, thus allowing higher dose delivery compared to an external beam.[8,9] Radioembolization has shown promising outcomes in primary and secondary liver malignancies in several studies. There are currently two types of radioembolization using Y90 microspheres — TheraSphere^®^ (MDS Nordion, Ottawa, Ontario, Canada) is made of glass, and SIR-Spheres^®^ (Sirtex Medical, Sydney, Australia) is made of resin. This review focuses on the indications, efficacy, side effects, and future direction of Y90 therapy using TheraSphere^®^ in patients with HCC.

## INDICATIONS

Therasphere^®^ can be used for radiation treatment or as a neoadjuvant to surgery or transplantation, in patients with inoperable HCC, who can have placement of appropriately positioned hepatic arterial catheters.[10] According to a consensus panel, report by the Radioembolization Brachytherapy Oncology Consortium, patients considered for radioembolization therapy would include those with (1) unresectable hepatic primary or metastatic cancer, (2) liver-dominant tumor burden, and (3) a life expectancy of at least three months.[11] It is also indicated for HCC patients with partial or branch portal vein thrombosis (PVT) / occlusion and studies have shown its safety and efficacy in patients with portal vein thrombosis.[12]

## EFFICACY

Comprehensive literature reviews have described the clinical and scientific evidence of Y90 therapy.[13,14] The consensus panel report mentioned earlier has concluded that there is sufficient evidence to support the safe and effective use of this locoregional therapy in HCC patients.

Few studies have been done on the use of TheraSphere^®^ in managing HCC. In a report by Kamel *et al*. on 13 patients, prospectively treated with TheraSphere^®^, magnetic resonance imaging was used to compare 24 hours pre-treatment and an average follow-up of 55 days post therapy.[15] There was a mean decrease in arterial enhancement of 22% and a mean decrease in venous enhancement of 25% in the targeted tumors, and unchanged tumor size in both targeted and non-targeted tumors. The median survival was reported as 12 months from the time of diagnosis.

In another study by Keppke *et al*., the response rates of 42 patients according to the World Health Organization (WHO), Response Evaluation Criteria in Solid Tumors (RECIST), necrosis and combined criteria (RECIST and necrosis) were 26, 23, 57, and 59%, respectively, after treatment with glass microspheres. The median survival for Okuda I patients was 660 days. The authors concluded that size criteria alone was not accurate in assessing tumor response after Y90 therapy and suggested that the imaging findings, using a combined criteria (size and necrosis), was more accurate.

Young *et al*., addressed the question of re-treatment using this therapy, by studying the relationship between the cumulative radiation dose and the development of liver toxicities in 41 patients, stratified to Okuda I and II.[16] They reported a statistically significant mean cumulative radiation dose of 390 Gy and 196 Gy tolerated by Okuda I and Okuda II patients, respectively, before toxicity occurred. This suggested that some patients were able to tolerate multiple treatments prior to the development of liver toxicities. The median survival from date of first treatment was 660 days and 431 days for Okuda I and Okuda II, respectively, (*P* = 0.44).

Kulik *et al*., reported that a group of 21 patients from a large database of 251 patients had undergone Y90 glass microsphere therapy and were subsequently bridged to transplantation.[17] A majority of patients experienced toxicities, including fatigue. Mean alpha-fetoprotein (AFP) reduced by 33% from pre-treatment levels and 66% of the patients had complete necrosis by pathological examination. Disease recurrence occurred in four of 21 patients, with a mean time to recurrence of 250 days. The authors concluded that Y90 treatment achieved complete necrosis in a majority of targeted lesions, in patients bridged to transplantation, but recurrence was a possibility despite the radiographic findings of complete necrosis. Subsequently, Kulik *et al*., went on to report the safety of Y90 in a cohort of 108 patients treated with glass microspheres, with subset analyses evaluating differences in patients with and without PVT.[12] Here, they concluded that the microembolic effect of Y90 microspheres did not raise the risk of liver adverse events in patients with proven PVT. Glass microspheres did not result in a microembolic effect that is seen with other loco-regional therapies using larger diameter particles.

In a recent study by Hilgard *et al*., the authors concluded that Y90 glass microspheres were safe and effective for use, even in patients with a compromised liver function.[18] However, since the time to progression and survival were similar to systemic therapy in a group of patients with advanced HCC, they suggested that randomized controlled trials in combination with systemic therapy are needed.

## SIDE EFFECTS

The side effects of radioembolization include fatigue, nausea, anorexia, vomiting, fever, abdominal discomfort, and cachexia.[19,20] These are usually not serious enough to warrant hospitalization.

Hepatobiliary toxicity may occur and is assessed using liver enzymes and metabolite levels, that is, alanine transaminase (ALT), aspartate aminotransferase (AST), alkaline phosphatase, bilirubin, and albumin. However, it is difficult to conclude whether changes in these levels are brought about by worsening hepatic cirrhosis or because of the radioembolization procedure. Hepatic toxicity has been reported to be as high as 20%[16] and can lead to morbidity and mortality.

Severe complications such as ulceration can be caused by the spread of the microspheres to the gastrointestinal tract. This can be prevented by careful mapping of the blood vessels, to look for aberrant vasculature from the branches of the hepatic artery that supply the gastrointestinal tract.

Transcatheter Y90 radioembolization is an invasive procedure. Nevertheless, it has a very low possibility of causing vascular injury and is mostly seen in patients who are already on systemic chemotherapy.[21] This is because systemic chemotherapy causes weakening of the vessel wall, thereby increasing susceptibility to injury.

Radiation pneumonitis has been shown to occur when the lung shunt function (LSF) is greater than 13%.[22] The LSF is used to calculate the dose that would be administered to the lung, and an absolute contraindication to radioembolization is the predicted administration of a dose greater than 50 Gy, as a cumulative dose after multiple treatments.[10]

## FUTURE DIRECTIONS

Although several phase II studies have provided useful data, it is necessary to carry out randomized controlled trials to compare TheraSphere^®^ therapy with the conventional care for HCC patients. This will enable the establishment of radioembolization as a universally accepted first line therapy for inoperable HCC. The advent of targeted molecular therapy is a new era in HCC treatment. Thus, it would also be useful if clinical investigations look into the safety and toxicity effects of combining cytotoxic Y90 therapy and the cytostatic mechanism of targeted therapies. This will in turn facilitate an improved clinical outcome and overall survival.

## CONCLUSIONS

Radioembolization has been established to have an important role in the management of liver tumors. It has the capability to improve survival and quality of life of HCC patients. The mild adverse events after radioembolization rarely require hospitalization. More serious adverse events can be minimized by careful selection of patients, using accepted dosimetry models, and employing meticulous technique. There are currently no randomized clinical trials done on TheraSphere^®^ and none of the studies so far have shown a survival benefit. Thus, although it represents a very promising therapy with excellent initial results, it cannot be fully recommended yet till well-designed, large, randomized clinical studies are conducted, showing survival benefits of TheraSphere^®^ therapy. These studies will help to further support the use of TheraSphere^®^ as a safe and efficacious treatment in the management of liver tumors.
